# Clinical Experience of Using Telemedicine for the Management of Patients Using Continuous Subcutaneous Insulin Infusion in a Highly Complex Latin American Hospital

**DOI:** 10.3390/ijerph20095719

**Published:** 2023-05-04

**Authors:** Guillermo Edinson Guzman, María Fernanda Escobar, Oriana Arias-Valderrama, María Angélica Guerra, Veline Martínez

**Affiliations:** 1 Fundación Valle del Lili, Departamento de Endocrinología, Cali 760032, Colombia; guillermo.guzman@fvl.org.co; 2 Departamento Medicina, Facultad de Ciencias de la Salud, Universidad Icesi, Cali 760031, Colombia; 3 Fundación Valle del Lili, Departamento de Telemedicina, Cali 760032, Colombia; 4 Fundación Valle del Lili, Centro de Investigaciones Clínicas, Cali 760032, Colombia; 5 Fundación Valle del Lili, Departamento de Medicina Interna, Cali 760032, Colombia

**Keywords:** telemedicine, telemedicine services, insulin infusion bump, continuous glucose monitoring, diabetic adults, glycemic control

## Abstract

Introduction: Continuous subcutaneous insulin infusion (CSII) has emerged as a potential solution for diabetes management during the pandemic, as it reduces the need for in-person visits and allows for remote monitoring of patients. Telemedicine has also become increasingly important in the management of diabetes during the pandemic, as it allows healthcare providers to provide remote consultations and support. Here, we discuss the implications of this approach for diabetes management beyond the pandemic, including the potential for increased access to care and improved patient outcomes. Methods: We performed a longitudinal observational study between 1 March 2020 and 31 December 2020 to evaluate glycemic parameters in diabetic patients with CSII in a telehealth service. Glycemic parameters were time in range (TIR), time above range, time below range, mean daily glucose, glucose management indicator (GMI), and glycemic variability control. Results: A total of 36 patients were included in the study, with 29 having type 1 diabetes and 6 having type 2 diabetes. The study found that the proportion of patients achieving target glucose variability and GMI remained unchanged during follow-up. However, in patients with type 2 diabetes, the time in target range increased from 70% to 80%, and the time in hyperglycemia decreased from 2% to 0%. Conclusions: The results of this study suggest that telemedicine is a strategy for maintaining glycemic control in patients using CSII. However, the lack of access to the internet and adequate telemonitoring devices make it difficult to use on a large scale in emerging countries like ours.

## 1. Introduction

Diabetes mellitus (DM) is a chronic and complex disease with global distribution and increasing prevalence [1]. According to the International Diabetes Federation (IDF), there were approximately 537 million people with diabetes worldwide in 2021. From 2000 to 2023, the IDF has reported that there has been a significant increase in the prevalence of diabetes globally. In the year 2000, there were about 151 million people with diabetes worldwide, which means that in the last 23 years the number of people with diabetes has more than tripled [2], and in Colombia, diabetes affects approximately 1,676,885 patients [3]. Despite advances in the study of the disease, the development of drugs, and new technologies to manage it, there are still many barriers to achieving goals and improving diabetes outcomes [4,5]. In response to basal–bolus failure in T1DM/T2DM patients, continuous subcutaneous insulin infusion (CSII) has been an effective strategy [6,7].

During the COVID-19 pandemic, DM was the second most prevalent comorbidity among patients with severe and critical disease manifestations [8]. Telehealth services and teleconsultation have become fundamental tools for the monitoring, treatment, and follow-up of patients with various medical conditions [9]. At the same time, the pandemic forced major changes in healthcare delivery, notably, the rapid shift from face-to-face care to telehealth, in order to minimize exposure to infection while providing access to needed healthcare services [10], and telemedicine emerged as an alternative to continue providing outpatient medical consultation services [11].

Fundación Valle del Lili (FVL) is a highly complex teaching hospital in Latin America that provides high-complexity health services to the population of southwestern Colombia. In March 2020, driven by lockdown measures due to the COVID-19 pandemic, a telemedicine service was implemented in FVL, to continue the follow-up of patients from different specialties, including patients diagnosed with DM. This study aims to describe glycemic control in patients with type 1 and 2 diabetes mellitus (T1DM and T2DM) managed with a CSII throughout the endocrinology follow-up with the telemedicine program implemented in our institution.

## 2. Materials and Methods

### 2.1. Study Design

In a single center, we performed an observational study of glycemic control in T1DM and T2DM patients managed with CSII followed by telemedicine during the COVID-19 health emergency between 1 March 2020, and 31 December 2020. This study was conducted according to the Declaration of Helsinki guidelines; and was classified as a risk-free study according to resolution No. 008430 of 1993, article 11, numeral A of the Ministry of Health and Social Protection of Colombia. The protocol of this study was approved by the Ethics Committee of the Fundación Valle del Lili (Protocol No. 1752; Act No. 155-2021). This study did not require intervention or intentional modification of the biological, physiological, psychological, or social variables of the participants; therefore, no informed consent was necessary.

### 2.2. Population

Participants were over 18 years of age with type 1 and type 2 diabetes who had been diagnosed with diabetes for more than one year, had at least two controls by teleconsultation in the period mentioned using continuous glucose monitoring (CGM) more than 70% of the time, and had failed previous medical treatment, i.e., their diabetes did not reach control goals despite multiple management with oral medication and they had to be adherent to their insulin management and use a CSII for at least 3 months. All patients who met the inclusion criteria were invited voluntarily to participate in the study and only those who accepted were included. They received diabetes education from the diabetes team and a specialist educator and attended to their primary care center for exams. Exclusion criteria were: patients with incomplete teleconsultation records; women who were pregnant or planning to become pregnant; patients in another clinical trial; patients with active malignancy, visual impairment, or any disability that affected their ability to use the insulin pump; and nonadherence to treatment.

Participants used the Medtronic Paradigm Veo Enlite sensor (Northridge, CA, USA), Medtronic MiniMed 640G Enlite sensor (Northridge, CA, USA) and Medtronic Minimed 670G using Guardian™ Sensor 3 (Northridge, CA, USA).

### 2.3. Variables

Information collection about comorbidities, parameters of CSII, clinical test at the time of the first teleconsultation as glycosylated hemoglobin, creatinine, lipid profile parameters, and variation delta were included in the following consultations. Time in range (TIR), time above range, time below range, mean daily glucose, glucose management indicator (GMI), glycemic variability control, admission to the emergency room for an event related to glycemic control, hospitalization, and death were recorded.

### 2.4. Data Sources

The variables were collected from the medical records generated in the teleconsultation. The information corresponding to the glycemic controls was derived from downloading the CSII data from the web page provided by Medtronic (Carelink^TM^, Brampton, ON, Canada) before the appointment. The data were registered into the digital database (BDClinic, Cali, Colombia) by a trained member of the research team, while another unrelated researcher performed quality audits by comparing random information with medical records.

### 2.5. Telemedicine Program Overview

Patients completed at least 2 virtual consultations (initial and follow-up). Initially, patients received education from the diabetes team composed of a nutritionist, diabetes nurse and endocrinologist, where they were taught how to use the insulin pump, how to use the CareLink platform for recording glucose monitoring data, and how to download glucose data.

Telemedicine follow-up was performed using the Microsoft Teams platform (Microsoft Corp., Redmond, WA, USA). In the first teleconsultation, the physician verified clinical conditions, ongoing treatment, and adherence to recommendations on healthy lifestyle changes. The recorded glycemia data were monitored, and treatment changes were made as needed. At the second teleconsultation, which was performed after at least 30 days, glycemic control, and changes from the first consultation were measured. All of the information collected during the teleconsultations was recorded in an electronic medical record.

### 2.6. Statistical Analysis

A descriptive analysis of the qualitative variables was made according to frequency and proportion. For the quantitative variables, normality was checked using the Shapiro–Wilk test. If normally distributed, the mean and standard deviations are presented as the median, and interquartile ranges are presented if nonnormally distributed. Chi-square and Student’s t tests were calculated between the first and second teleconsultations. Data were analyzed using STATA version 15.0 software (StataCorp LLC, College Station, TX, USA).

## 3. Results

A total of 109 patients with a diagnosis of diabetes were users of CSII, all participants were invited to participate in the study, but only 36 patients who met the selection criteria voluntarily decided to accept. The median age of T1DM patients was 29 years compared to 50 years in T2DM patients. The median time from diagnosis was higher in T1DM patients, and this group presented more microvascular complications. Minimed 640 was the most common CSII, followed by Minimed 670G. The median follow-up on T1DM was 3 months vs. 6 months in T2DM. Table 1 shows the general characteristics of the population.

Generally, glycemic parameters were similar after teleconsultation (see Table 1). In T1DM, time below (TBR), in range (TIR), and above (TAR) were similar. However, in T2DM patients, TIR increased from 70 (63–83) to 82 (78–89) mg/dL (*p* = 0.01), and TAB decreased from 180–250 mg/dL from 18 (11–23) to 11 (5–14) mg/dL (*p* = 0.003). Additionally, the proportion of patients achieving goals of consensus statement targets in T1DM was reduced for <5% of time >250 mg/dL 24 (83%) to 20 (69%) (*p* = 0.003). In T2DM, <4% of the time, between 54–70 mg/dL was reduced in 4 (57%) to 3 (42%) and 1% of time <54 mg/dL 4 (57%) to 3 (42%) (see Table 2).

During follow-up, HBA1c, microalbuminuria, and lipid profile did not show statistically significant differences (see Table 3). One patient required consultation in the emergency department and was hospitalized due to a hyperglycemic crisis. Both groups had no complications for T2DM and no deaths (see Table 4).

## 4. Discussion

Chronic disease incidence is increasing, and we need tools to contact and control our patients. COVID-19 was a challenge for health care systems and became a global crisis [11]. The high number of patients diagnosed with diabetes and obesity and the impact of SARS-CoV-2 infection have reflected the need to control these pathologies better as they significantly increase mortality and morbidity [11]. To prevent the spread of this virus and to conduct a strict follow-up of these chronic diseases (that can aggravate the outcomes), the design of new health care models is a priority [12]. Telemedicine allows for maintaining contact with patients, breaking down barriers to health care access. We show that remote follow-up can be a strategy even in a developing country.

Telemedicine is a technological tool that the World Health Organization (WHO) defined as “the provision of health services (where distance is a critical factor) by all health professionals, using information and communication technology for the exchange of valid information for the diagnosis, treatment, and prevention to promote the health of individuals and communities” [13]. It has been determined that telemedicine contributes to the fulfillment of the Sustainable Development Goals (SDGs), reduces the saturation of health centers, and decreases health gaps and inequities [14]. In 2010, a legislation was established in Colombia; it sets the guidelines for the development of telehealth and determines the parameters for the practice of telemedicine. However, it was not until the arrival of the COVID-19 pandemic that the use of telemedicine tools was prompted and enhanced in the country to continue the provision of health services in different areas, due to the lockdown and physical distancing measures recommended and adopted in the country [15].

Telemedicine is a strategy for medical monitoring with favorable results in chronic diseases and a health care complement to facilitate accessibility and opportunity [16,17]. Monitoring diabetic patients through telehealth has improved diabetes control, decreasing glycosylated hemoglobin [17]. Parise et al. reported the data of 166 patients with DMT1 under management with CGM, attended by teleconsultation, and they evidenced a significant improvement in the time in range (TIR) and the absence of diabetic ketoacidosis or severe hypoglycemia requiring hospitalization [18]. Viñals et al. also reported a significant improvement in TIR, TAR 180–250 mg/dL, and TAR > 250 mg/dL during follow-up with teleconsultation [19].

CSII coupled with glucose monitoring systems has advanced to improve glycemic control results [20]. This has been implemented with good results in the type 1 and type 2 Diabetic patients, reducing HbA1C and the frequency of hypoglycemia, positively affecting the quality of life of the patients. Since the implementation of this therapy, it has been evolving from insulin micro-infusion pumps to semi-automatic hybrid insulin delivery systems, which has allowed ostensible improvements in the glycemic control of patients. In our report, we included different types of technology, from the Minimed VEO system to Minimed 670G. Real-life studies show that TIR 70–180 can be in a minimum of 640 g 62.98% ± 13.97% and in a minimum of 670 g 72.44% ± 9.5%, which confirms that new models improve TIR [21]. Stone et al. conducted a study on real-world glucose outcomes after 3 months of MiniMed 670G use [21]. Data were extracted from the CareLink™ system (Medtronic, Northridge, CA, USA), the database to which Medtronic users upload their pumps and/or CGMs to view their diabetes management history over a given period. After analyzing the CareLink data, one study showed an increased time range from an average across all age groups of ~62.5% in Manual Mode to ~70.8% in Auto Mode. Our control experience is superior, showing a time in the baseline range of 79% (70–84%) and no change over time despite remote monitoring independent of the technology used, even though only 25% of the population was using the most recent 670G model that uses Auto mode.

The type of diabetes is not a limitation to define if a patient benefits from a CSII [22]. Studies in real life with Minimed 670G confirm these findings with TIR 70–180 of 73.47 ± 9.40 vs. 74.50 ± 12.15 in DM1 and DM2, respectively [19]. In our experience, TIR should be kept in teleconsultation. However, significant reductions were evidenced in the proportion of patients with T1DM who achieved >70% of time 70–180 mg/dL and <0.5% of time >240 mg/dL as well as in T2DM patients who achieved <4% of time <70 mg/dL and <1% of time <54 mg/dL with no change in the overall, without affecting TAR, TIR, TBR, HBA1c or complications. Our good results in TIR could be explained by the multidisciplinary support that includes an endocrinologist and a diabetes educator. Additionally, downloading data from the Carelink system allows us to evaluate glycemic control and device adherence with assertive communication. Previously, the Carelink system was associated with improved glycemic control in children with type 1 diabetes on CSII [22].

Hypoglycemia is quite frequent in the diabetic population [23]. CSII has been successful in the reduction of hypoglycemic episodes [20,24]. Mesa et al. demonstrated the association between the use of CGM and the decreased frequency of hypoglycemic episodes (severe and non-severe) compared to glucometers at home [19]. In our study, TBR was similar in T1DM and T2DM. Nobody needed a visit to the emergency room for hypoglycemia during follow-up. COVID-19 confinement period was a risk factor for developing diabetes and for the increase of diabetes-related complications [25], whether it was hyperglycemia or hypoglycemia. A study found a significant increase in physical inactivity, sitting time during the day, greater consumption of food, and an unhealthy eating pattern [26], which is favored by the fact that being at home provides greater access to food and snacks, and they eat as a coping mechanism for stress and lack of interest in cooking [27]. Smart applications and systems that are designed to help individuals with diabetes manage their insulin pumps can be particularly helpful in this context, as they can provide real-time feedback and guidance on dietary choices, exercise regimens, and insulin dosing [28]. However, as noted, the effectiveness of these tools can be limited by factors such as poor access to the internet and inadequate devices for telemedicine in developing countries. It is important to work on individual strategies to manage glycemic control. In our case, patients with CSII had follow-up with a nutritionist, which may have been a favorable factor.

In addition, another frequent complication of diabetes that causes hospitalization is hyperglycemic crises. Additionally, the prevention of these crises is a remote patient follow-up objective. Parise et al. evaluated the effectiveness of follow-up in two virtual visits; no hospitalization for ketoacidosis was reported [18]. During our follow-up, only one patient was hospitalized for a hyperglycemic crisis, diabetic ketoacidosis type. This event was precipitated by urinary sepsis that required hospitalization in the intensive care unit with a favorable outcome. The results obtained showed that patients with diabetes were well controlled prior to the pandemic and maintained adequate management during and after the pandemic thanks to telemedicine follow-up, it proved to be an effective way to monitor their condition and achieve good control and treatment. Additionally, it helped to decrease the number of complications as access to healthcare services was guaranteed. Continuous monitoring through telemedicine can provide several additional benefits. For example, telemedicine monitoring can allow for early detection of any changes in a patient’s glycemic control, which can help prevent serious complications in the future. In addition, continuous monitoring through telemedicine can help patients receive support and education regarding their disease, which can be especially helpful for those living in rural or remote areas with limited access to medical care [23]. Further work is required to determine long-term sustainability and support.

However, not all research obtains satisfactory results with telemonitoring. Ghosal et al. created a simulation model to predict the impact of confinement due to COVID-19 on diabetes and related complications. They evidenced a significant increase in HbA1c during the follow-up period, thereby calculating a proportional annual increase in the rate of diabetes-related complications, which increased as the length of confinement increased [29]. However, our results were optimal, but we did not identify variables associated with reasonable glycemic control in remote follow-up. Nevertheless, multidisciplinary support and access to a computer with internet are essential tools for this goal. In our case, 109 diabetic patients with CSII had at least one teleconsultation follow-up, but only 36 participants had a second follow-up with this method. Different reasons could be the cause.

Despite the many advantages of telemedicine, some barriers still limit its implementation and use, such as digital illiteracy, limited access to digital and telecommunication services mainly in rural areas, inherent limitations of telemedicine (e.g., the difficulty or lack of physical examination) [30]. Colombia has 50 million inhabitants; approximately 38 million people have access to mobile internet, only 8.4 million have access to fixed internet, and 60.7% of the population has no access to a computer [31], our main problem for the follow-up of telemedicine patients in Colombia is access to the internet and a computer for downloading data. In conclusion, telemedicine has many potential benefits in Colombia; there are also challenges that must be addressed in order to ensure that all patients have access to high-quality healthcare. It is important to work towards improving access to technology and ensuring that telemedicine services are equipped to provide comprehensive care to all patients.

### Strengths and Limitations

We evaluated the effectiveness of technology in a particular group of high-risk patients and the efficacy of teleconsultation intervention in this specific group of patients. The study was conducted in a Latin American population, in which diabetes mellitus plays an essential role due to its high prevalence. Although the sample size is small, Fundación Valle del Lili serves a heterogeneous group of patients representing the nuances of the country’s population (Indigenous, Mestizos, Blacks, Whites).

Our study has limitations. Due to our study’s retrospective, observational nature, only an association between the CGM data obtained in both periods (not causation) can be inferred from the results. We did not look over changes in daily life, such as diet and exercise, which may influence outcomes and results in glycemic and metabolic control. The data were obtained from two short periods (with some missing data), and we need to know whether positive findings can be extended over a longer time.

Further studies should have rigorous methods to measure the effects of an intervention on quality of life, well-being, and organizational issues (cost-effectiveness).

## 5. Conclusions

Our study suggests that telemedicine provides a safe strategy for monitoring and adjusting the management of diabetic patients using CSII. Despite this, barriers to telemedicine access need to be explored. Telemedicine could be a promising tool for further study; it was a safe strategy for monitoring and adjusting the management of chronic diseases such as diabetic patients using CSII.

## Figures and Tables

**Table 1:** Baseline characteristics of patients.

Feature	Type 1 Diabetes n = 29	Type 2 Diabetes n = 7	Overall n = 36
Age. years *	29 (25–34)	50 (44–64)	31.5 (27–44.5)
Female. n (%)	22 (75.83)	5 (71.43)	27 (75)
BMI *	24 (21.9–26.5)	25.6 (19.4–27.1)	
Educational level. n (%)			
High school	20 (68.9)	7 (100)	27 (75)
Professional	9 (31.0)	0	9 (25)
Time since diagnosis of diabetes. years *	15 (9–26)	13 (7–24)	
Macrovascular complications. n (%)			
No	29 (100)	6 (85.71)	35 (97.22)
Coronary disease	0 (0)	1 (14.29)	1 (2.78)
Microvascular complications. n (%)	13 (44.83)	0 (0)	13 (36.11)
No	16 (55.17)	7 (100)	23 (63.89)
Diabetic nephropathy	9 (31.0)	0 (0)	9 (25)
Diabetic neuropathy	5 (17.2)	0 (0)	5 (13.89)
Diabetic retinopathy	8 (27.59)	0 (0)	8 (22.22)
Oral antidiabetic drugs. n (%)	5 (17.2)	2 (28.5)	7 (19.44)
Type of insulin pump. n (%)			
1.Paradigma Veo	1 (3.45)	0(0)	1 (2.78)
2.Minimed 640	22 (75.86)	4 (57.14)	26 (72.22)
3.Minimed 670	6 (20.6)	3 (42.86)	9 (25)

Data are presented as median (IQR) *. BMI: Body Mass Index.

**Table 2:** Continuous glucose monitoring parameters during the teleconsultation.

	Type 1 Diabetes n = 29			Type 2 Diabetes n = 7			Overall n = 36		
	Teleconsultation			Teleconsultation			Teleconsultation		
Feature	First	Second	*p* Value	First	Second	*p* Value	First	Second	*p* Value
Mean sensor glucose concentration (mg/dL) *	137.5 ± 12.8	135.3 ± 10.1	0.40	138.5 ± 18.1	148.2 ± 15.7	0.10	137.72 ± 12.59	139.9 ± 12.67	0.357
GMI *	6.56 ± 0.29	6.53 ± 0.24	0.59	6.6 ± 0.4	6.8 ± 0.38	0.82	6.57 ± 0.32	6.61 ± 0.29	0.55
Coefficient of variation (%) *	32.4 ± 4.14	32.2 ± 4.3	0.06	34.2 ± 6.12	35.8 ± 5.0	0.64	32.52 ± 4.68	33.57 ± 4.21	0.10
Time < 54 mg/dL (%) **	0	0 (0–1)	NA	0	0.5 (0–1)	NA	1 (0–1)	0 (0–1)	NA
Time 54–70 mg/dL (%) **	5 (2–7)	3.5 (1–6)	0.55	4 (2–6)	6.5 (3–8)	0.42	4 (2–5)	3 (1–5)	0.52
Time in range 70–180 mg/dL (%)) **	79 (72–86)	77.5 (72–86)	0.36	70 (63–83)	82 (78–89)	0.01	79 (70–84)	79 (72–86)	0.20
Time 180–250 mg/dL (%) **	15.1 ± 7.6	15.5 ± 7.2	0.65	19 ± 9.5	10.5 ± 5.9	0.00	15 (10–22)	14 (9–18)	0.69
Time > 250 mg/dL (%) **	1 (0–3)	1 (0–3)	0.01	2 (0–7)	0 (0–2)	0.25	1 (0–4)	1 (0–3)	0.12
The proportion of patients achieving consensus statement targets. n(%)									
<7% GMI	17 (59%)	15 (52%)	0.70	3 (42%)	3 (42%)	0.25	20 (55%)	18 (50%)	1.00
<36% of CV	16 (55%)	13 (45%)	0.12	2 (28%)	3 (42%)	1.00	18 (50%)	16 (44%)	0.69
>70% of time 70–180 mg/dL	20 (69%)	18 (62%)	0.02	3 (42%)	5 (71%)	0.27	23 (63%)	23 (63%)	0.69
<25% of time 180–250 mg/dL	23 (79%)	19 (66%)	0.06	4 (57%)	6 (85%)	NA	27 (75%)	25 (69%)	0.41
<5% of time >250 mg/dL	24 (83%)	20 (69%)	0.00	5 (71%)	5 (71%)	0.12	29 (80%)	25 (69%)	0.56
<4% of time <70 mg/dL	14 (48%)	15 (52%)	0.88	4 (57%)	3 (42%)	0.01	18 (50%)	18 (50%)	0.39
<1% of time <54 mg/dL	14 (48%)	14 (48%)	0.19	4 (57%)	3 (42%)	0.01	18 (50%)	17 (47%)	0.51

Data are presented as the mean ± SD *, median (IQR) **. GMI: glucose management indicator CV: coefficient of variation.

**Table 3:** Laboratory parameters in the first and second teleconsultations.

	Type 1 Diabetes n = 29			Type 2 Diabetes n = 7			Overall n = 36		
	Teleconsultation			Teleconsultation			Teleconsultation		
Feature	First	Second	*p* Value	First	Second	*p* Value	First	Second	*p* Value
Glycosylated hemoglobin%	7.18 ± 0.71	7.0 ± 0.7	0.391	7.31 ± 0.92	7.2 ± 0.6	0.743	7.2± 0.8	7.08 ± 0.13	0.351
Cholesterol LDL mg/dL	103.1 ± 27.5	113 ± 45.1	0.416	135.5 ± 39.6	80 ± 21.1	0.213	114.56 ± 13.54	105.67 ± 12.27	0.537
Cholesterol HDL mg/dL	62.9 ±15.2	58.9 ± 11.6	0.222	66.5 ± 16.1	10.4 ± 9.5	0.879	66.83 ±4.47	61.83 ±3.20	0.288
Triglycerides mg/dL	85.2 ± 69.0	112 ± 65.4	0.133	74.5 ± 26.4	111.8 ± 62.9	0.258	70.77 ±6.53	111.23± 19.36	0.045
Creatinine mg/dL	0.82 ± 0.19	0.73 ± 0.12	0.47	0.86 ± 0.10	0.98 ± 0.08	0.102	0.83 ± 0.04	0.82 ±0.05	0.751
Albuminuria mg/G	13.6 ± 20.2	19.1 ± 24.5	0.854	11.1 ± 10.2	7.94 ± 10.5	0.298	18.54 ± 9.14	18.52 ±9.14	0.993

Data are presented as the mean (SD).

**Table 4:** Complications.

Feature	Type 1 Diabetes n = 29	Type 2 Diabetes n = 7	Overall n = 36
Emergency admission, n (%)	3 (10.3%)	0	3 (8.3)
Need for hospitalization, n (%)	3 (10.3%)	0	3 (8.3)
Hyperglycemic crisis, n (%)	1 (3.4%)	0	1 (2.8%)
Infections, n (%)	1 (3.4%)	0	1 (2.8%)
Death, n (%)	0	0	0 (0%)

Data are presented as N (%).

## Data Availability

The data used to support the findings of this study are restricted by Fundación Valle del Lili Ethics Committee to protect patient privacy. Data are available from Guillermo E. Guzmán for researchers who meet the criteria for access to confidential data.
